# Development of Bioorthogonal Reactions and Their Applications in Bioconjugation

**DOI:** 10.3390/molecules20023190

**Published:** 2015-02-16

**Authors:** Mengmeng Zheng, Li Zheng, Peiyuan Zhang, Jinbo Li, Yan Zhang

**Affiliations:** Institute of Chemistry & BioMedical Sciences, School of Chemistry and Chemical Engineering, Nanjing University, Nanjing 210093, China; E-Mails: zhengmengmeng453@163.com (M.Z.); ndzhengli@163.com (L.Z.); zpy1994121@163.com (P.Z.)

**Keywords:** bioorthogonal reactions, bioconjugation strategies, biomolecules, chemical probes

## Abstract

Biomolecule labeling using chemical probes with specific biological activities has played important roles for the elucidation of complicated biological processes. Selective bioconjugation strategies are highly-demanded in the construction of various small-molecule probes to explore complex biological systems. Bioorthogonal reactions that undergo fast and selective ligation under bio-compatible conditions have found diverse applications in the development of new bioconjugation strategies. The development of new bioorthogonal reactions in the past decade has been summarized with comments on their potentials as bioconjugation method in the construction of various biological probes for investigating their target biomolecules. For the applications of bioorthogonal reactions in the site-selective biomolecule conjugation, examples have been presented on the bioconjugation of protein, glycan, nucleic acids and lipids.

## 1. Introduction

Biological processes in living systems are extremely complicated but also highly required to be unveiled for biological and biomedical research. To study the molecular details of biological processes, active biological probes toward these processes are needed, for which various bioconjugation strategies have been developed to construct these probes [1]. Monoclonal antibodies and genetic fluorescent protein fusions represent typical biological strategies for bioconjugation and elucidation on the roles of specific proteins in dynamic cellular processes. However, other biomolecules such as glycans, lipids and nucleic acids were limited to study due to the relatively large size of fluorescent proteins and the low cell membrane permeability of antibodies. Over the past decade, the development of bioorthogonal chemistry strategy has shown great promise in overcoming these limitations.

Bioorthogonal reactions are chemical reactions that can proceed in biological systems without interaction with the inside biomolecules or interference on the whole system [2,3]. The ideal complementary components of bioorthogonal reactions should be inert and nontoxic to the biological systems, but highly selective and reactive with each other when present in the biological environment. The employment of bioorthogonal reactions as bioconjugation strategies will address the limitations coming with traditional biological strategies and allow detailed investigation on various specific biomolecules. To this end, several bioorthogonal reactions such as Staudinger ligation, click reaction, tetrazine ligation, and photo-click reaction have been developed and widely applied in bio-labeling [3,4,5]. Bioconjugation employing a bioorthogonal reaction typically involves a two-stage strategy: first, the introduction of one reactive component into a biomolecule (chemically or biochemically), followed by bioorthogonal conjugation to label the biomolecule with fluorophores or affinity tags.

In this review paper, we will summarize the often used bioorthogonal reactions and their development, followed by introduction on their applications in bioconjugation of proteins, glycans, nucleic acids and lipids.

## 2. Bioorthogonal Reactions and Recent Development

Bioorthogonal chemistry has found impacted applications in the field of chemical biology. In the past decade, a limited number of reactions including Staudinger ligation, click reaction, tetrazine ligation and photo-click reaction have been well developed, which have also been widely utilized for labeling biomolecules in the context of living cells and whole organisms. In this section, we will summarize the development of these bioorthogonal reactions.

### 2.1. Staudinger Ligation

**Scheme 1:**
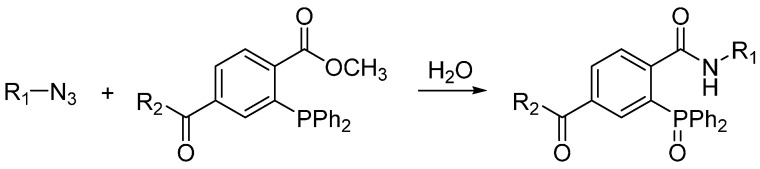
Staudinger ligation.

The Staudinger ligation was the first example of bioorthogonal ligation reaction. Its prototype reaction in which azides are transformed into primary amines upon reducing by phosphines was reported by Staudinger in 1919 and was first called the Staudinger reduction [6]. Bertozzi and co-workers modified the phosphine reagent to contain an electrophilic trap, the aza-ylide intermediate then reacts with the electrophilic ester carbonyl group and generates a stable amide bond after hydrolysis in water (Scheme 1) [7]. Shortly after the report on Staudinger ligation, traceless Staudinger ligation were reported, which the final amide-linked product is freed from the phosphine oxide moiety. The application of Staudinger ligation enabled selective biomolecule labeling not only in living cells but also in living animals [8,9]. However, the easy oxidation of phosphine reagents in the biological systems and slow kinetics of the Staudinger ligation remain as unsolved problems and obstacles for *in vivo* bioconjugation.

### 2.2. “Click” Reactions

#### 2.2.1. Copper-Catalyzed Click Reaction 

**Scheme 2:**
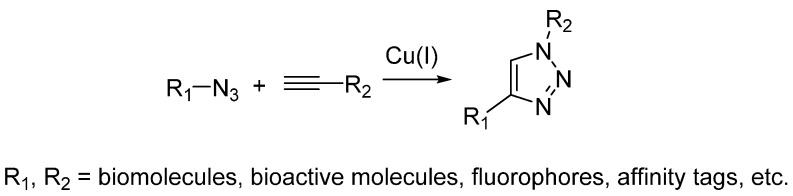
Copper-catalyzed alkyne-azide cycloaddition (CuAAC).

Up to now, the most commonly used and famous bioorthogonal reaction is the “click” reaction, which is the cycloaddition reaction between azides and alkynes. The prototype reaction was first reported by Michael in 1890s [10] and then well studied by Huisgen in the last century [11]. While, this conventional cycloaddition reaction is far away from being developed into bioorthogonal reaction due to its slow kinetics and harsh reaction conditions. In early 2000s, Sharpless and Meldal independently reported that this reaction could be dramatically accelerated by Cu(I) catalysis in aqueous solution [12,13]. This is the well-known “click” chemistry and named as copper-catalyzed alkyne-azide cycloaddition (CuAAC) (Scheme 2), which has been widely used as bioconjugation strategy in the field of chemical biology [14,15]. Nevertheless, the toxicity of Cu(I) makes the CuAAC not biocompatible enough and hinders their applications in living cells. To address this issue, several ligands, which can increase the reactivity of Cu(I) and thereby reduce the amount of Cu(I) to decrease the toxicity, have been recently developed [3]. The use of ligands broadened the biological applications of CuAAC and allowed live cell imaging [16,17].

#### 2.2.2. Strain-Promoted Click Reaction

**Scheme 3:**
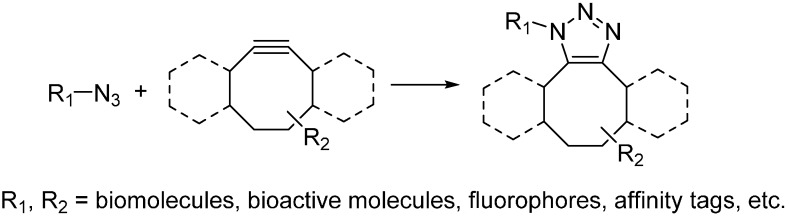
Strain-promoted alkyne-azide cycloaddition (SPAAC).

To avoid using the toxic copper catalyst, Bertozzi used strained cyclooctynes instead of linear alkynes [18]. The alkyne in strained form makes it highly reactive and can undergo cycloaddition with azide quickly under physiological environment. This reaction is named as the strain-promoted alkyne-azide cycloaddition (SPAAC) or copper-free click reaction (Scheme 3) and has been widely used for bioconjugation in not only living cells but also living animals [19,20]. While, poor water solubility of cyclooctynes limits their applications in biological environment. Chemical modification on the cyclooctynes has been demonstrated as a way to partially improve their solubility. In addition, the substitutions on cyclooctynes are also critical, which will determine the second-order rate constants of SPAAC. Bertozzi has systematically investigated the possible substitutions on cyclooctynes and found difluorinated cyclooctyne showed higher reactivity [21]. Boons developed biarylazacyclooctynone by fusing two benzene rings to cyclooctyne, whose second-order rate constant was approximately three times higher than that of simple cyclooctyne [20]. In the meanwhile, van Delft enhanced the reactivity of simple cycloctyne by introducing an amide bond into the ring, which could easily be synthesized in high yields [22]. van Delft also developed bicyclo[6.1.0]nonyne as another cyclooctyne analog for click chemistry, which also possessed high reactivity toward azide [23].

**Scheme 4:**
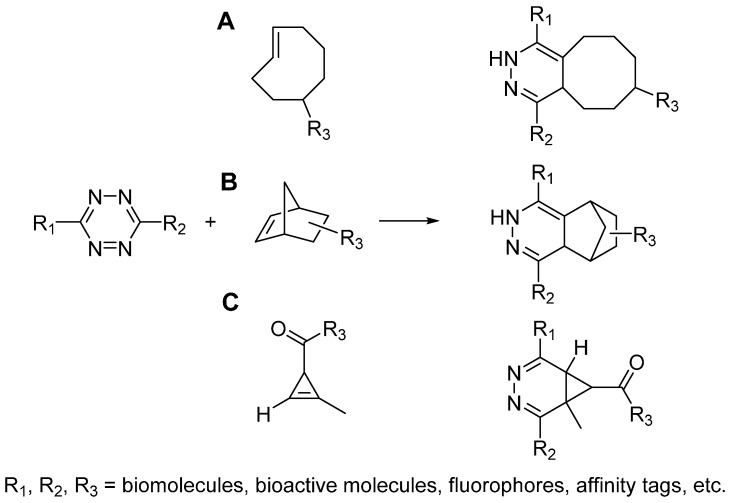
Tetrazine ligations between tetrazine and trans-cyclooctene (**A**), norbornene (**B**), cyclopropene (**C**).

### 2.3. Tetrazine Ligation

The prototype reaction of tetrazine ligation was first reported by Sauer in 1990s, which was the cycloaddition reaction between tetrazine and alkene [24,25,26]. In 2008, Fox and Hilderbrand independently reported the bioorthognal tetrazine ligations, which used trans-cyclooctene [27] (Scheme 4A) and norbornene [28] (Scheme 4B) respectively to react with tetrazine in aqueous solution. It is worth to note that the tetrazine ligation proceeds extremely fast, whose second-order rate constant is as high as 2000 M^−1^·s^−1^. With the employment of tetrazine ligation, successful pretargeted live cell imaging was achieved [28,29]. Considering the large size of *trans*-cyclooctene and norbornene may limit the application of this cycloaddition reaction, Prescher and Devaraj independently investigated the smallest strained alkene, cyclopropene, as the other component of tetrazine ligation (Scheme 4C) [30,31]. However, the cyclopropene is not stable enough, which may easily react with biomolecules or get polymerized. To address these issues, Prescher and Devaraj did different modifications on the cyclopropene ring, including adding methyl and carbamate moieties. Even though the second-order rate constants of reaction between cyclopropenes and tetrazine are lower (~0.1–13 M^−1^·s^−1^), it is still an ideal candidate reaction for bioconjugation and allows genetic coding of this small strained alkene into proteins or metabolic labeling of glycans [30,32].

### 2.4. Photo-Click Reaction

**Scheme 5:**
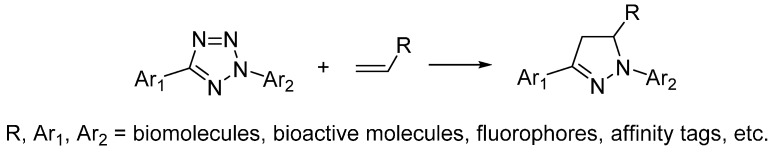
Photo-click reaction.

Inspired by the photoactivated cycloaddition reaction between 2,5-diphenyltetrazole and methyl crotonate reported by Huisgen in 1967 [33], Lin explored the cycloaddition reaction between tetrazole and alkene as bioorthogonal reaction in 2008, which is named as photo-click reaction (Scheme 5) [34,35,36]. The photoactivation of tetrazole will generate nitrile imine dipole, which will quickly undergo cycloaddition reaction with alkene and give nitrogen as the only byproduct. Noteworthily, the product of photoclick reaction, pyrazoline, is also fluorescent, making it an ideal bioorthogonal reaction for both ligation and labeling. In combination with genetic coding, tetrazole or alkene was inserted into the side chains of amino acids and protein labeling was then realized with the application of photo-click reaction [37,38]. However, the need of 365 nm UV light for photoactivation limits the application of photo-click reaction in biological systems. To overcome this limitation, Lin investigated a series of tetrazoles with different substitutions and found the oligothiopene-based tetrazoles that could be activated by 405 nm light [39]. Recently, Lin reported naphthalene-based tetrazoles could be activated by two photon excitation and the photoclick reaction could happen by 700 nm laser light activation [40]. The two-photon photoclick reaction allowed spatial and temporal imaging of live mammalian cells.

## 3. Applications of Bioorthogonal Chemistry for Biomolecule Bioconjugations

### 3.1. Applications of Bioorthogonal Chemistry for Protein Bioconjugations

Proteins are the most abundant biomolecules in living systems and participate in almost all the biological processes. So far, the introduction of bioorthogonal reporters into proteins, in combination with the bioorthogonal reactions, provides the most powerful tools for the exploration of protein expression, localization and function in their native environment. A variety of strategies have been developed to selectively incorporate the bioorthogonal chemical groups into the proteins of interest, including chemical or enzymatic modifications, residue-specific incorporation using the cell’s translational machinery and more precise strategy—site-specific incorporation by exogenously introduced aaRS-tRNA pair. The site-specific incorporation is more favorable as its less interference with the protein’s structure and function.

Labeling bioactive small molecules with alkynes, they turned into bioorthogonal probes and allowed target protein labeling inside cells and proteome profiling by performing click reactions [41], while with the incorporation of cyclopropene into bioactive small molecules, protein labeling by tetrazine ligation in living cells was achieved [42]. Direct incorporation of cyclopropene into protein through chemical modification, BSA was fluorescently labeled through tetrazine ligation [30]. The photo-click reaction was also used to directly label proteins *in vitro* through introduction of tetrazole group into proteins by chemical modification and fluorescent labeling of microtubules in living cells was achieved by labeling taxoid with tetrazole group and application of photo-click reaction [43].

**Scheme 6:**
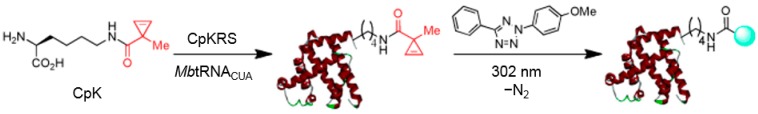
Site-specific labeling of proteins by genetic coding of cyclopropene and photoclick reaction. Reproduced from reference [38]. CpKRS, CpK-specific aminoacyl-tRNA synthetase; *Mb*tRNA_CUA_, *methanosarcina barkeri* tRNA(CUA).

By genetic coding expansion strategy, tetrazole and alkene was introduced into various proteins in cells and fluorescent labeling of these proteins was realized by photo-click reaction [35,37,44]. *Trans*-Cyclooctene and norbornene were introduced into proteins in *E. coli.* and mammalian cells by genetic coding, bio-labeling was also achieved by the tetrazine ligation [32,45]. The cyclopropene moiety has also been demonstrated as a bioorthogonal reporter for protein labeling by genetic coding and photo-click reaction (Scheme 6) [38]. Except using bioorthogonal reactions for protein labeling, these bioconjugation strategies have also been extended to investigate biological processes. For instance, the azide modified lysine was incorporated into a bacterial secretion factor OspF, which allowed detection of OspF’s secretion in bacterial extracellular space by click reaction [46]. The incorporation of bioorthogonal functionalities into pathogens allowed investigation on these proteins during pathogenesis. Using similar strategy, bioorthogonal reporters were also introduced on the surface of live hepatitis D virus and bio-labeling under living conditions was realized by click reaction for the first time [47]. By incorporation of azide groups into an acid-stress chaperone HdeA in *E. coli.* and combination of click reaction, pH value inside *E. coli.* was determined successfully [48]. In addition, tetrazine ligation not only could be used for bio-labeling but also for chemical decaging. By introducing trans-cyclooctene into side chain of lysine and combining genetic coding, the resulted protein inside living cells could react with tetrazine, inducing release of modified moieties on the side chain of lysine and consequent activation of this protein [49].

### 3.2. Application of Bioorthogonal Chemistry for Glycan Bioconjugations

Glycan is another particularly attractive target for bio-imaging as it plays key roles in numerous biological processes. For example, sialic acid containing glycoconjuates are closely related with bacterial infection and viral invasion [50,51]. Aberrant glycosylation is thus implicated in progression of various diseases including cancers. Probing the dynamic changes of glycan is then of great importance for investigating glycobiology and improving disease diagnosis and therapeutics. Classic methods for monitoring glycans rely on molecular recognition with probe bearing lectins or antibodies, which are limited due to the poor tissue permeability and toxicity.

Unlike proteins, glycosylation is a posttranslational modification and the imaging strategies based on genetically encoded tags are not applicable for visualizing glycans. Alternatively, imaging of glycans is usually achieved by metabolic glycan labeling. In general, monosaccharide building blocks functionalized with bioorthogonal chemical tags (Table 1) can be incorporated into target glycans via the cell’s own metabolic machinery [52]. After installing the bioorthogonal group into glycans on cell surface, a complementary imaging probe is then conjugated to the glycans via bioorthogonal reaction, enabling the visualization of the tagged glycoconjugates.

**Table 1:** The often used monosaccharide with bioorthogonal chemical tags.

Natural Monosaccharide	Chemical Report Bearing Monosaccharide
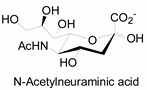	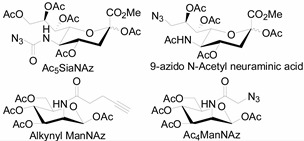
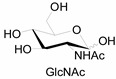	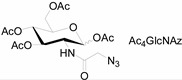
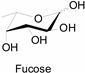	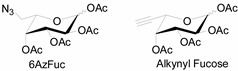
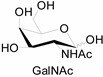	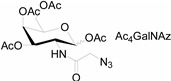

**Scheme 7:**
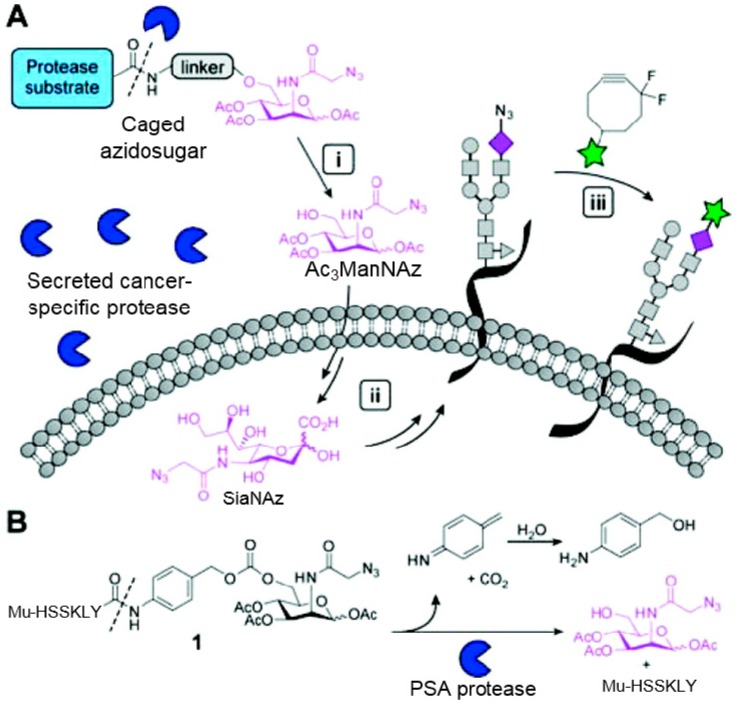
(**A**) Strategy for tissue-specific glycan labeling via protease activation. (i) Ac3ManNAz was released by protease cleavage of caged azidosugar. (ii) The azidosugar was then metabolized into cell-surface glycans. (iii) The azide-modified glycans were then fluorescently labeled through SPAAC; (**B**) Cleavage process of caged azidosugar. Reproduced from reference [54] (an open-access article by ACS author choice. This is an unofficial adaptation of an article that appeared in an ACS publication. ACS has not endorsed the content of this adaptation or the context of its use).

In 2000, azide was metabolized into cell-surface glycans through N-azidoacetylmannosamine (Ac4ManNAz) by Bertozzi, and Staudinger ligation with a biotinylated triarylphosphine was then performed to form stable adducts on the cell surface [7]. This is the first example of glycan labeling through bioorthogonal chemistry. Then, glycans in living mice were successfully labeled by injecting Ac4ManNAz into mice and performing Staudinger ligation *ex vivo* [9]. In 2008, N-azido-acetylgalactosamine (Ac4GalNAz) was used by Bertozzi to metabolic incorporate azide into living zebrafish and *in vivo* imaging of glycans in developing zebrafish was achieved by performing click reaction [53]. Although the metabolic labeling approach has made great progress in glycan labeling, it is still not selective to abnormal cells and will cause glycan labeling in most tissues. To achieve specific labeling, Bertozzi used a caged metabolic precursor that could be activated by prostate-specific antigen (PSA) (Scheme 7) [54]. PSA could cleave its substrate and released the active azidosugar. Therefore, the metabolic labeling only happened where PSA was present, realizing specific labeling and imaging of glycans on the cell surface by SPAAC. Besides using enzymatic activation, Chen used liposome functionalized with targeting ligands to deliver azidosugars and the targeting ability of ligands realized cell-specific metabolic glycan labeling by CuAAC [55]. In addition, the metabolic labeling of glycans by CuAAC has been successfully utilized to study glycome during cardiac hypertrophy, highlighting the potential of bioorthogonal chemistry in revealing pathological processes in living systems [56].

### 3.3. Application of Bioorthogonal Chemistry for Nucleic Acids Bioconjugations

Nucleic acids including DNA and RNA are essential units for cellular biological processes and studying nucleic acids in their native environment is thus of great importance in the field of chemical biology. Nucleotides and their analogs could be incorporated into the genomes of replicating cells by endogenous enzymes, which has made direct labeling of nucleotides the most commonly used method for nucleic acid detection [57]. Metabolic labeling of DNA has traditionally been performed using [^3^H]thymidine or BrdU, which requires autoradiography, or DNA denaturation and antibody staining [58,59]. However, the detection of BrdU is limited by the poor tissue permeability of the BrdU antibodies. The emergency of bioorthogonal labeling methods provides valuable tools to study the biological macromolecules in their native environment.

**Scheme 8:**
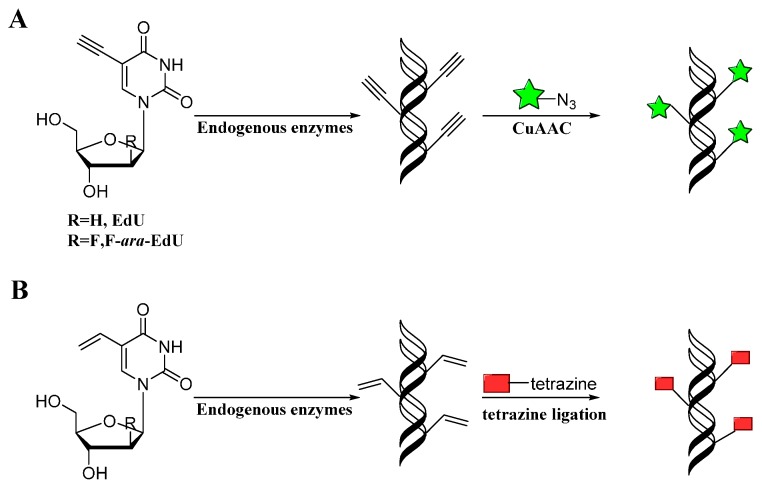
Labeling DNA with alkyne (**A**) or alkene (**B**) modified nucleotides through CuAAC or tetrazine ligation, respectively.

**Figure 1:**
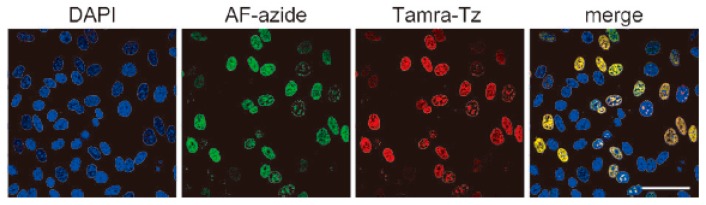
Cellular DNA were imaged simultaneously by VdU and EdU, reproduced from reference [63]. DAPI stained cellular nucleus; AF-azide stained alkyne-modified DNA; Tamra-Tz stained alkene-modified DNA; Merge means the overlay of these images. Scale bar, 50 µm.

Mitchison developed a method for labeling DNA *in vivo* based on the incorporation of 5-ethynyl-2-deoxyuridine (EdU) into cellular DNA during DNA replication, the ethynyl groups of EdU could be imaged by performing CuAAC with fluorescent azides (Scheme 8A) [60]. This method does not require sample fixation or DNA denaturation. Furthermore, EdU can be used to detect cell proliferation in large, fresh tissue and organ explants. Noteworthily, this EdU unit was commercialized by Invitrogen and applied in studying cell proliferation and differentiation [61]. However, EdU was proved to be toxic that can lead to DNA instability and cell-cycle arrest. Luedtke then used F-ara-EdU as metabolic labels instead of EdU (Scheme 8A) [62]. F-ara-EdU is less toxic than EdU and exhibited selective DNA labeling, and had less effects on genome function. Besides, Luedtke utilized tetrazine ligation as an alternative bioconjugation strategy for DNA imaging [63]. They synthesized 5-vinyl-2'-deoxyuridine (VdU), which is selectively metabolized into cellular DNA, where it was imaged by using tetrazine ligation (Scheme 8B). Then, VdU and EdU were incorporated into cellular DNA simultaneously. CuAAC and tetrazine ligation were performed in parallel for dual-labeling of cellular DNA (Figure 1), showing these bioorthogonal methods for detection or imaging of VdU and EdU are both efficient and orthogonal. In addition, a new DNA building block bearing the diaryltetrazole unit as the photoactivatable group was used to label cellular DNA with photo-click reaction, which allowed spatial and temporal imaging of DNA [64].

5-hydroxymethylcytosine (5-hmC) constitutes the sixth base in the mammalian genome and has potential applications as epigenetic marker. He developed an efficient and selective bioorthogonal labeling method to differentiate 5-hmC from 5-methylcytosine (5-mC) and determined the distribution of 5-hmC in mammalian genomes [65]. β-Glucosyltransferase (β-GT) was utilized to transfer a chemically tagged glucose, 6-N_3_-glucose, onto 5-hmC, for bioorthogonal labeling. Then 5-N_3_-hmC was labeled with biotin through click reaction and the attached biotin moiety can serve as a tag for detection, enrichment and sequencing purposes. This method may help to show the dynamics of 5-hmC in living cells, and the strategy may help learn more cellular behaviors related to 5-hmC.

### 3.4. Application of Bioorthogonal Chemistry for Lipids Bioconjugations

Lipids play critical biological roles in regulating many vital biological pathways and pathophysiological events, such as enforcing protein membrane binding and attaching lipids onto protein [66]. In these biological processes, lipids play key roles as ligands and substrates. Many experimental methods have been developed for their functional analysis: chromatography and mass spectrometry (MS), radioactive labeling and fluorescent lipid derivatives. However, their analysis is complicated due to their extremely complex and dynamic behavior. The development of bioorthogonal reactions has facilitated the study of lipid activities by providing the ability to selectively label lipids bearing bioorthogonal tags within complex biological samples. The bioorthogonal tags are incorporated into lipids by metabolism of their biosynthetic precursors and provide the means to image these biomolecules within their native environments.

Alkyne labeled choline lipids were metabolically incorporated into phospholipids and the resulted lipids were selectively and sensitively labeled in cells through CuAAC [67]. The bioorthogonal lipids allowed direct imaging of phospholipid synthesis, turnover and their localization inside cells. Similarly, phospholipid derivatives were pre-labeled with strained alkyne functionalities. After incorporation into cells, direct imaging of phospholipids in both fixed cells and living cells were achieved by SPAAC [68]. By adding azides and photo-crosslinking moieties into lipids, proteins interacted with lipids were investigated in *Saccharomyces cerevisiae* [69]. After photo-crosslinking, proteins interacted with lipids in the inner mitochondrial membranes were biotinylated through SPAAC and further identified by mass spectrometry. By using active lipids that can covalently interact with phospholipases, activity-based probes in which fluorophosphates modified with alkynes were synthesized [70]. With the active probes, phospholipases were labeled with fluorescent dye through CuAAC and a novel phospholipase was successfully identified.

Protein fatty-acylation is fundamental and critical in biological processes, which is also limited to study due to the lack of methods for protein lipidation. By combining Staudinger ligation, fatty-acylated proteins in mammalian cells were detected and characterized with azide modified fatty acids [71]. And by functionalization fatty acids with azide or alkyne tags, these chemical reporters were then metabolically incorporated into mammalian cells [72,73]. Proteins related to these fatty acids were then labeled with fluorescent dyes through CuAAC. These chemical probes were then further utilized in *E. coli* to identify bacterial lipoproteins that interacted with fatty acids, allowing sensitive imaging and large-scale discovery of unknown lipoproteins in gram-negative bacteria [74].

## 4. Outlook

Up to now, only a limited number of bioorthogonal reactions have been developed. Except the mostly used bioorthogonal reactions described above, other reactions such as Pd-mediated coupling reaction and cycloaddition reaction between quinone methide and vinyl thioether have also been recently developed for bioconjugation [75,76,77,78,79]. van Delft also recently developed another type of strain-promoted click reaction, which used nitrones instead of azides and was then named as strain-promoted alkyne nitrone cycloaddition (SPANC) [80]. The application of these bioorthogonal reactions has allowed direct protein labeling inside cells or target identification of bioactive small molecules. Nevertheless, the biocompatibility of these chemical tags such as toxicity, selectivity, sensitivity and stability when they are present in biological environment still needs further improvement. For example, the toxicity of Cu and Pd prevents them from applications in living cells. Even though ligands of these metals could partially solve this problem, these reactions are still not bioorthogonal enough. In addition, it is impossible to use catalysts for animal study. Therefore, development of bioorthogonal reactions without requirement for catalysts will benefit further biological studies. Tetrazine ligation and SPAAC could proceed without catalyst, while the stability of these reactants in biological systems is not good enough. Besides, the bioorthogonality of the components and reaction sensitivity of these bioorthogonal reactions are still the major obstacles for *in vivo* bioconjugations. Improvements in current bioorthogonal reactions are still needed to broaden their applications. In addition, chemical probes with multi-functionality are usually needed for detailed studies of complicated biological processes, in which orthogonal bioconjugation strategies are needed [81]. Then, new bioorthogonal reactions complementary to current bioorthogonal reactions are still needed for construction of multifunctional probes, which may be discovered by high-throughput screening [82]. With the development of bioorthogonal reactions, chemical probes will become more powerful and biological processes will be revealed with the application of these probes.
